# Reviewer acknowledgement 2012

**DOI:** 10.1186/1471-244X-13-85

**Published:** 2013-04-22

**Authors:** Deesha A Majithia

**Affiliations:** 1 BioMed Central, Floor 6, 236 Gray’s Inn Road, London, WC1X 8HB, UK

## Abstract

**Contributing reviewers:**

The editors of *BMC Psychiatry* would like to thank all our reviewers who have contributed to the journal in Volume 12 (2012).

## 

Argelinda Baroni

USA

Lena Sanci

Australia

Joao Apostolo

Portugal

Allison Webel

USA

Dennis Hofman

Netherlands

Ilanit Hasson-Ohayon

Israel

Narendra Nath Sarkar

India

Isabelle Carrard

Switzerland

John Eastwood

Australia

Luis Agüera

Spain

Thomas Kallert

Germany

Fredrik Spak

Sweden

Cheryl Carmin

USA

Greg Carter

Australia

Triptish Bhatia

India

Heather Stuart

Canada

John Joska

South Africa

Mike Firn

United Kingdom

Byung-Joo Ham

Korea, South

Sue Wilson

United Kingdom

Jesse Cougle

USA

Thomas Ehring

Netherlands

Jamie Ringer

USA

Chris Hawley

United Kingdom

Claude P. Muller

Luxembourg

Gioia Mura

Italy

Harald Gündel

Germany

Mark Opler

USA

Daniel Dillon

USA

Michael Gerhard Odenwald

Germany

George Parker

USA

Clement C. Azodo

Nigeria

Lukasz Cichocki

Poland

Roland Weierstall

Germany

Antoine Pariente

France

Michelle Lim

USA

Emanuel Severus

Germany

Scott Lamont

Australia

Fikru Tesfaye

Ethiopia

Susan Rees

Australia

Robert David Schweitzer

Australia

Kirsi Ahola

Finland

Acebo Garcia Guerrero

Spain

Matthias Liechti

Switzerland

Shannon Wiltsey Stirman

USA

Salvador Guinjoan

Argentina

Michael Fitzgerald

Ireland

David Vago

USA

Alv A. Dahl

Norway

Elisabeth Schramm

Germany

Jeffrey Fuller

Australia

Wojciech Baranowski

Poland

Rami Bou Khalil

Lebanon

E Fuller Torrey

USA

Bronwyn Kaiser

USA

Dianne Vella-Brodrick

Australia

Kazuyuki Shinohara

Japan

James Tapp

United Kingdom

Kirsten Müller-Vahl

Germany

Matt Muijen

Denmark

Michael Witthöft

Germany

Josep Antoni Ramos-Quiroga

Spain

Thomas Rigotti

Germany

Robin Emsley

South Africa

Shane Shucheng Wong

USA

Jie Zhang

USA

Amy Morgan

Australia

Esther Williamson

United Kingdom

Niki Antypa

Italy

Kristin Schneider

USA

Bayard Roberts

United Kingdom

Derrick Silove

Australia

Audra Ames

USA

Georg Juckel

Germany

Alexandra Martin

Germany

Angus Macbeth

United Kingdom

Qun Mai

Australia

Timo Partonen

Finland

Martin Härter

Germany

Aartjan Beekman

Netherlands

Matthew Hotopf

United Kingdom

Matthew Sunderland

Australia

Kylie Dingwall

Australia

James Gold

United States Minor Outlying Islands

Jorge Cervilla

Spain

Marco Chiesa

United Kingdom

Morten Hesse

Denmark

Martyn Harling

United Kingdom

Willem-Paul Brinkman

Netherlands

Margaret Jones

United Kingdom

Katherine Sharkey

USA

Anwar Mechri

Tunisia

Frank Neuner

Germany

Giulia Perini

Italy

Ilse Maria Johanna Van Beljouw

Netherlands

Rosa M. Baños

Spain

Tadashi Takeshima

Japan

Carolina Escobar

Mexico

Regina P. Markus

Brazil

Sameer Jauhar

United Kingdom

Carolyn Dewa

Canada

Keming Gao

USA

Andelko Vidovic

Croatia

Svein Friis

Norway

Aristotle Voineskos

Canada

Tomas Kasparek

Czech Republic

Claire Marnane

Australia

Tracey Varker

Australia

Gavin Brown

New Zealand

Ladislav Hosak

Czech Republic

Frank Schneider

Germany

Matthias Jaeger

Switzerland

Jitender Sareen

Canada

Kerstin Weber

Switzerland

Hsien-Yuan Lane

Taiwan

Christopher Baethge

Germany

Julia Louw

South Africa

Mark Boschen

Australia

Robert Barkin

USA

Tracy Greer

USA

Chi-Yung Shang

Taiwan

Niina Markkula

Finland

Aimee Hunter

USA

Wolfram Kawohl

Switzerland

Liangsuo Ma

USA

Patrick Mcgowan

Canada

Antonio Preti

Italy

Ulrich Hegerl

Germany

Rene Ernst Nielsen

Denmark

Sandra Dittmann

Germany

Alisa O’Riley

USA

John Read

New Zealand

Steffen Moritz

Germany

Lars Kessing

Denmark

Siri Thoresen

Norway

Roseanne Armitage

USA

Sophia Vinogradov

USA

Peter Van Der Velden

Netherlands Antilles

Doris Nilsson

Sweden

Maurizio Pompili

Italy

Marloes Gerrits

Netherlands

Andreas Menke

Germany

Joy Duxbury

United Kingdom

Dorotea Muck-Seler

Croatia

Nora Alsageer

Yemen

Stephanie Keller

USA

Joëlle Micallef

France

Daniel Eduardo Vigo

Argentina

David Goodman

USA

Huanguang Jia

USA

Amit Shrira

Israel

Ragnhild Orstavik

Norway

Fernanda Serpeloni

Germany

Thomas Olino

USA

Durisala Desaiah

USA

Harald Baumeister

Germany

Ulrich W. Preuss

Germany

Adelaide Robb

USA

Heinz Grunze

United Kingdom

Gianfranco Spalletta

Italy

Jessica Hellings

USA

Barna Konkoly Thege

Canada

Sarah Knowles

United Kingdom

Ben Jansen

USA

Jonathan Shaffer

USA

Binit Shah

USA

Didier Schrijvers

Belgium

Joseph Lee

Australia

Omar Sery

Czech Republic

Svein Bergvik

Norway

Supriya Syal

South Africa

Holger Sorensen

Denmark

Jan Spijker

Netherlands

Michael Thase

USA

Jamie Fernandez

USA

Steven Dilsaver

USA

Lianne Kurina

USA

C Sanmartin

Spain

Pratap Sharan

India

Enrique Baca-Garcia

Spain

Michiaki Nagai

Japan

Stefan Borgwardt

Switzerland

Josep Garre-Olmo

Spain

Marina Kukla

USA

Nicola Reavley

Australia

Timothy Crow

United Kingdom

Marco Menchetti

Italy

Johan Fastbom

Sweden

Masayo Uji

Japan

Tara Donker

Australia

Lijie Wu

China

Lora Beebe

USA

Jason Uslaner

USA

Laura Meis

USA

Stephan Heres

Germany

Anne Wand

Australia

Cindy Eckart

Germany

Arifulla Khan

USA

Amelia Aldao

USA

Bo Netterstrøm

Denmark

Kristine Chapleau

USA

Vasant Hirani

United Kingdom

Gerasimos Kolaitis

Greece

Steven Dworkin

USA

William Spaulding

USA

Rudolf Uher

United Kingdom

Carmen Garcia-Pena

Mexico

Juan Li

China

Clement Hamani

Canada

Sophie Walsh

Israel

Cornelis Mulder

Netherlands

Bekh Bradley

USA

Brian Marx

USA

Judith Bernstein

USA

Vikas Duvvuri

USA

Birgitte Thylstrup

Denmark

Edle Ravndal

Norway

Majed Ashy

USA

Viviane Kovess

France

Roland Mergl

Germany

Pietro Giusti

Italy

John Davis

USA

Raffaella Calati

Italy

Robert Göder

Germany

Michele Raja

Italy

Mark Van Der Gaag

Netherlands

Janne Tolstrup

Denmark

Liesje Donkin

Netherlands

Marie-Cecile Bralet

France

Massimo Pasquini

Italy

Annet Kleiboer

Netherlands

Wakako Umene-Nakano

Japan

Carroll W. Hughes

USA

Srinivasan Tirupati

Australia

Jiska Peper

Netherlands

Neeltje Van Haren

Netherlands

Jess Fiedorowicz

USA

Jenny Yiend

United Kingdom

Thomas Berger

Switzerland

Tom Burns

United Kingdom

Matteo Balestrieri

Italy

Cecilia Sighinolfi

Italy

Hidetoshi Takahashi

Japan

Paul Yip

Hong Kong

Andres Neuhaus

Germany

Michael Anestis

USA

Matthew Large

Australia

Fonseca Pedrero

Spain

Sheila Hardy

United Kingdom

Saba Kassim

United Kingdom

Rowena Jacobs

United Kingdom

Francisco Diaz

USA

Jürgen Hoyer

Germany

Ulf Malm

Sweden

Ruslan Leontjevas

Netherlands

Luca Lavagnino

Italy

Sandy Simpson

Canada

Maite Ferrin

Spain

Angela Wagner

USA

Yiru Fang

China

Reinhold Kilian

Germany

Keith Lloyd

United Kingdom

Julio Sánchez-Meca

Spain

Bernardo Carpiniello

Italy

Patricia Quinn

USA

Mohammadreza Mokhtari

USA

Peter Britton

USA

Guillermo Gonzalez-Burgos

USA

Angela Hassiotis

United Kingdom

Michael Freeman

USA

Kerri Clough-Gorr

USA

Barbini Barbara

Italy

Yuri Milaneschi

Netherlands

Sheri Johnson

USA

Tilman Steinert

Germany

Chong Guan Ng

Malaysia

Terri Henderson

Spain

Andrea Eugenio Cavanna

United Kingdom

Bryan Bernard

USA

Philippe Conus

Switzerland

Jochen Mutschler

Switzerland

Pier Paolo Pani

Italy

Kia-Chong Chua

United Kingdom

Mauro Giovanni Carta

Italy

Ruth Benca

USA

Gianluca Serafini

Italy

Barbara J. Burns

USA

Mukaila Raji

USA

Catherine Harmer

United Kingdom

Yaara Zisman-Ilani

Israel

David Kimhy

USA

David Ndetei

Kenya

Gregory Zaric

Canada

Lucy Burns

Australia

Walter Heinrichs

Canada

Uwe Wollina

Germany

Rebecca Monk

United Kingdom

Philipp Kuwert

Germany

Francesco Saverio Bersani

Italy

Hywel Williams

United Kingdom

Caroline Vandeleur

Switzerland

Davy Vancampfort

Belgium

Michiko Nakazato

Japan

Ignacio Jáuregui-Lobera

Spain

Martine Flament

Canada

Felice Iasevoli

Italy

Yuming Ning

USA

Carlos Garcia Forero

Spain

Gerhard Dammann

Switzerland

Catherine Harrington

USA

Russell Schachar

Canada

Hiroyuki Uchida

Japan

Maria Kalafati

Greece

Katja Wingenfeld

Germany

Carl Anderson

USA

Michaela Schwarzbach

Germany

Anna Rita Atti

Italy

Alaptagin Khan

USA

Diana Koszycki

Canada

Fredrik Ahs

USA

Amin Muhammad Gadit

Canada

Fernando Fernandez-Aranda

Spain

Urs Hepp

Switzerland

Bradley Gaynes

USA

Patricia Van Oppen

Netherlands

Valdo Ricca

Italy

Colin Martin

United Kingdom

Jaana Suokas

Finland

Richard Josiassen

USA

Heidi Lyneham

Australia

Mark Zimmerman

USA

Alessa Von Wolff

Germany

Masaki Kondo

Japan

Sofia Brissos

Portugal

Alan Prossin

USA

Per-Anders Rydelius

Sweden

Mark Jordans

Nepal

Jeffrey Burke

USA

Joseph Mcguire

USA

Benjamin Buck

USA

Louanne Davis

USA

Marianna Mazza

Italy

Dorly Deeg

Netherlands

Ingo Schäfer

Germany

Rikke Jørgensen

Denmark

Matthew Kurtz

USA

Wojciech Zareba

USA

Ad Kerkhof

Netherlands

Stuart Lee

Australia

Kuowei Tay

Australia

Kerstin Ekberg

Sweden

Chiho Yatsuga

Japan

Hirotaka Kosaka

Japan

Jennifer Rose

USA

Stefano Ferracuti

Italy

Hadass Goldblatt

Israel

Manote Lotrakul

Thailand

Philip Sugarman

United Kingdom

Francesca Martino

Italy

Norio Mori

Japan

Sian Jones

United Kingdom

Junichi Furusho

Japan

Massimo Mauri

Italy

Samuel Law

Canada

Maria Tillfors

Sweden

Abraham Rudnick

Canada

Paul Emmelkamp

Netherlands

Hans Kroon

Netherlands

Maroesjka Van Nieuwenhuijzen

Netherlands

Chin-Bin Yeh

Taiwan

Marijn Alice Prins

Netherlands

Elie Cheniaux

Brazil

Bruce Kinon

USA

Monnica Williams

USA

Silvia Bernardi

USA

Robert Ferrari

Canada

Andrea Roberts

USA

Edmond Teng

USA

Eric Storch

USA

Anne-Kathrin Fett

Netherlands

Guy Chouinard

USA

Martijn Kikkert

Netherlands

Toshihiko Satoh

Japan

Mulusew Gerbaba

Ethiopia

Young-Hoon Ko

Korea, South

Alfredo Rodriguez Munoz

Spain

Monica Gammelgard

Finland

Seiji Hama

Japan

Louise Michele Howard

United Kingdom

Shakeh Moamrtin

Australia

Estrella Montoya

Netherlands

Yehuda Pollak

Israel

Tara Donker

Netherlands

Su Yeon Lee

USA

Xu-Jie Zhou

China

Murat Kuloglu

Turkey

Michael Farrell

Australia

Martin Walter

Germany

Silvia Gibellini Manetti

Switzerland

Bita Moghaddam

USA

Johan Verwoerd

Netherlands

Kim Van Zoonen

Netherlands

Nanda Nj Rommelse

Netherlands

Paolo Girardi

Italy

Brian Bishop

Australia

Bonnie Wm Siu

Hong Kong

Anette Kersting

Germany

Amanda Wickett-Curtis

USA

Richard Adams

USA

Laura Sirri

Italy

Birgit Kleim

Switzerland

Arjen Noordhof

Netherlands

Hiroshi Nagayama

Japan

Tingzhong Yang

China

Soham Al Snih

USA

Gavin Lambert

Australia

Siow Ann Chong

Singapore

Gayle Doherty

United Kingdom

David Crockford

Canada

John Burnham

USA

Ina Beintner

Germany

Andrea Norcini Pala

Italy

Miguel Roca

Spain

Vivian Kafantaris

USA

Shivarama Varambally

India

Ryan Klein

Canada

Eling D. De Bruin

Switzerland

Patrik Roser

Germany

Margaret Niznikiewicz

USA

Stefan Priebe

United Kingdom

Takeshi Fujii

Japan

Nalini Sehgal

USA

Kelly Buck

USA

Ron Salomon

USA

Haralambos Tsorbatzoudis

Greece

Wim Van Brakel

Netherlands

Kristy Dalrymple

USA

Rohan Ganguli

Canada

Elske Salemink

Netherlands

Beate M. Schulze

Switzerland

Massimo Casacchia

Italy

Gabriel Coutinho

Brazil

Megan Grant

USA

Philip Yanos

USA

Jen Vohs

USA

Derek Fisher

Canada

Ravi Bhat

Australia

Borko Jovanovic

USA

Kyunghee Lee

Korea, South

Natacha Carragher

Australia

Yoko Kamio

Japan

Yasuyuki Okumura

Japan

Anna Van Meter

USA

Marc B.J. Blom

Netherlands

Helge Frieling

Germany

Martin Teicher

USA

Toshio Munesue

Japan

Vicente Molina

Spain

Georita Frierson

USA

Srihari Gopal

USA

Alfred Lange

Netherlands

Claudi Bockting

Netherlands

Mark Powers

USA

Changsu Han

Korea, South

Luigi Rocco Chiri

Italy

Joanna Leaviss

United States Minor Outlying Islands

Isobel Cameron

United Kingdom

Norbert Hermanns

Germany

Qingrong Tan

China

Stephen Stansfeld

United Kingdom

Sebastian Walther

Switzerland

Abebaw Fekadu

Ethiopia

Brian Miller

USA

Jennifer Teller

USA

Damien Leger

France

Majid Ghayour

Iran

Antje-Kathrin Allgaier

Germany

Sebastian Voigt-Radloff

Germany

Edmund Silins

Australia

Matthew Hoptman

USA

Katy Robjant

United Kingdom

R. Andrew Chambers

USA

Hanspeter Moergeli

Switzerland

Magdalena Paczkowski

USA

Anders Hakansson

Sweden

Mari Strand

Norway

Mario Louza

Brazil

Paul Moran

United Kingdom

J J Sandra Kooij

Netherlands

Hiroaki Hori

Japan

Matthias Berking

Germany

Samantha Outcalt

USA

Hubertina Maria Bronzwaer

Netherlands

Yasser Khazaal

Switzerland

Heather Brenhouse

USA

Youngtae Cho

Korea, South

José Luis Ayuso-Mateos

Spain

Douglas Zatzick

USA

Viviane Leal

Brazil

Karin Jensen

USA

Betul Baykan

Turkey

Egil Nygaard

Norway

Eric Vermetten

Netherlands

Carolina Blaya

Brazil

Murat Gulsun

Turkey

David Gavin

USA

Seth Gillihan

USA

Ghulam Soomro

United Kingdom

Marcus Sokolowski

Sweden

Jan Froelich

Germany

Sean Perrin

United Kingdom

Patricia Casey

Ireland

Diego Moliner-Urdiales

Spain

Leonardo Fontenelle

Brazil

David L Cutler

USA

Cinnamon Bidwell

USA

David Roe

Israel

Luis Gonzalo De La Casa

Spain

Heinz Grunze

United Kingdom

Clement Zai

Canada

Leonard Schalkwyk

United Kingdom

Bregje Van Spijker

Australia

Secundino López-Pousa

Spain

Martine Bouvard

France

Michael Vitacco

USA

Luis Salvador-Carulla

Spain

Akira Babazono

Japan

Beverley Raphael

Australia

Kazuhito Yokoyama

Japan

Alessandro Serretti

Italy

Scott Weich

United Kingdom

Maria Carmela Tartaglia

Canada

Stacie Dusetzina

USA

Ralph Kupka

Netherlands

Frank R Sharp

USA

Sabrina Grondhuis

USA

Ronald Kessler

USA

Charles Reynolds

USA

Yael Caspi

Israel

Ling Zheng

USA

Paul Gorczynski

Canada

Rajeshwari Punekar

USA

Tine Nordgreen

Norway

I-Chia Chien

Taiwan

Yamashita Hiroshi

Japan

Toshihiko Matsumoto

Japan

Gesine Grande

Germany

Kia Faridi

Canada

Solvig Ekblad

Sweden

Joanna Bredski

United Kingdom

Stephen Levine

Israel

Claire Advokat

USA

Arnaud Duhoux

Canada

Zahava Solomon

Israel

Eva Velthorst

Netherlands

Michelle Keightley

Canada

Bryan Mccormick

USA

Gilles Lafargue

France

Raffaele Popolo

Italy

Nicole Vogelzangs

Netherlands

Michael Leathley

United Kingdom

Stewart Agras

USA

Wen-Jun Gao

USA

Candice Boyd

Australia

Mehdi Tafti

Switzerland

Anja Wittkowski

United Kingdom

Ana Adan

Spain

Pieter-Jan Carpentier

Netherlands

John Monahan

USA

Borwin Bgandelow

Germany

Jordan Silberman

USA

Andrea Fiorillo

Italy

Jane Fisher

Australia

Nassir Ghaemi

USA

Aaron Salinas

Mexico

Susanne Knappe

Germany

Gwenole Loas

France

Radi Masri

USA

Rafael De Sousa

Brazil

Amelia Gulliver

Australia

Juliana Diniz

Brazil

Nicholas Mitchell

Canada

Geertje Goedhart

Netherlands

Evelyn Bromet

USA

Ying Zhang

Australia

Sylvain Grignon

Canada

Hossein Hassanian-Moghaddam

Iran

James Dillon

USA

Steffi G. Riedel-Heller

Germany

Franz Resch

Germany

Aysegul Ozerdem

Turkey

Liliana Dell’Osso

Italy

Gitta Lubke

USA

Ricardo Carrion

USA

Eduard Kraft

Germany

Brett Mcdermott

Australia

Arianna Di Florio

United Kingdom

Richard Hermann

USA

Gabriele Pitschel-Walz

Germany

Lin-Yang Chi

Taiwan

Fermin Mayoral

Spain

Izabela Barbosa

Brazil

Shirli Werner

Israel

Douglas Faries

USA

Jan Volavka

USA

Jiang.Li@Northwestern.Edu Li

USA

Martien Kas

Netherlands

Ioannis Michopoulos

Greece

Sophie Davison

Australia

Clement Francois

France

Gabor Gazdag

Hungary

Constantin Tranulis

Canada

Takefumi Suzuki

Japan

Britta Bernhard

Germany

Jochen Hardt

Germany

Andrew Papanicolaou

USA

Alex Cohen

United Kingdom

Michael Chmielewski

Canada

Daniel O’Connor

Australia

Robin Kok

Netherlands

Mary C Smith Fawzi

USA

Jose De Leon

USA

Kiyoto Kasai

Japan

Kevin Antshel

USA

Jill Littrell

USA

Klaus Lieb

Germany

Arno Van Dam

Netherlands

Jeffrey Bridge

USA

Masoud Kamali

USA

Jamie Micco

USA

Chih-Ken Chen

Taiwan

Shahrzad Mavandadi

USA

Corine De Ruiter

Netherlands

Helen Davies

United Kingdom

Amir Tal

Israel

Miao-Yu Tsai

Taiwan

Ines Kaufmann

Germany

Carryl Navalta

USA

Lorenzo Tonetti

Italy

David Scott

Australia

Mary Johnson

USA

Norio Sugawara

Japan

Shuai-Ting Lin

Taiwan

Maria Panagioti

United Kingdom

Stephanie Burns

USA

Gavin Andrews

Bahamas

Ulrik Fredrik Malt

Norway

Santino Gaudio

Italy

Ann Polcari

USA

Jeronimo Saiz-Ruiz

Spain

Traolach Brugha

United Kingdom

Alberto pbiaghi

Italy

Belinda Liddell

Australia

Sudipto Chatterjee

India

Massimiliano Buoli

Italy

Haya Ascher-Svanum

USA

Dorothea Isele

Germany

Anne Berghofer

Germany

James Mcgough

USA

Danny Horesh

Israel

Jean-Christophe Chiem

Belgium

Daniel Taylor

USA

Stephen Wood

United Kingdom

Richard Musil

Germany

Markus Kösters

Germany

Atsurou Yamada

Japan

Lisanne Warmerdam

Netherlands

Mark Shevlin

United Kingdom

Anita Bengtsson-Tops

Sweden

Eric Rassin

Netherlands

Matthias Halldorsson

Iceland

Guilherme Polanczyk

Brazil

Kenneth Morrison

Canada

Brian Odlaug

USA

K.S. Jacob

India

Manfred Fichter

Germany

Darin Dougherty

USA

Henricus Van

Netherlands

Taro Kishi

Japan

Hanneke Creemers

Netherlands

Donna Ames

USA

Joanne Riebschleger

USA

William Bobo

USA

Ganesan Venkatasubramanian

India

Yasuhiro Kaneda

Japan

Dan Iosifescu

USA

Nicole Praschak-Rieder

Austria

Tony Merry

United Kingdom

Timothy Bolt

United Kingdom

Markus Moessner

Germany

Marcella Bellani

Italy

Ratilal Lalloo

Australia

Elisabeth Schauer

Italy

Sophia Frangou

United Kingdom

Pim Cuijpers

Netherlands

Cherrie Galletly

Australia

Jens Knud Larsen

Denmark

Amy Price

USA

Yoshio Minabe

Japan

Pascale Guicheney

France

Jaime Derringer

USA

Mitchell Byrne

Australia

Angela Nickerson

USA

Magda Tsolaki

Greece

Junichi Iga

Japan

Moli Paul

United Kingdom

Napoleon Waszkiewicz

Poland

Blake Dear

Australia

Eva-Lotta Brakemeier

Germany

Jocelyn Catty

United Kingdom

Joseph Pierre

USA

Mehul Trivedi

USA

Goran Arbanas

Croatia

Ross Norman

Canada

Simone Farrelly

United Kingdom

Einar Thorsteinsson

Australia

Clive Long

United Kingdom

Stefan Leucht

Germany

Hannie Comijs

Netherlands

Sandeep Grover

India

Eva Brandl

Canada

Colin Haile

USA

Isabel Krug

Australia

Giovanni Abbate-Daga

Italy

Nematollah Jaafari

France

Satoshi Takada

Japan

Nori Takei

Japan

Gloria Arankowsky-Sandoval

Mexico

Mairead Dolan

Australia

Ann Faerden

Norway

Samir Kumar Praharaj

India

Dieter Naber

Germany

Mari Hysing

Norway

Stephen Read

USA

Alec Davidson

USA

Justin Barry-Walsh

New Zealand

Lyndon Riviere

USA

Alexander Mcfarlane

Australia

Rajeev Krishnadas

United Kingdom

Carsten Wotjak

Germany

## Pre-publication history

The pre-publication history for this paper can be accessed here:

http://www.biomedcentral.com/1471-244X/13/85/prepub

